# 

**Published:** 1876-03

**Authors:** 


### Contents of March Number.

### ORIGINAL.

ART. I.—The Etiology of Elephantiasis, (Grecorum and Arabum,) Endemic Hæmaturia and Chyluria. By Chas. H. Richmond, M. D., .......... 281
ART. II.—Exercise as a Remedial Agent. By H. R. Hopkins, M. D., ..... 289
ART. III.—Ligation of the Carotid Artery for Injury. By E. T. Dorland, M. D., .......... 298
ART. IV.—Buffalo General Hospital. Notes of Surgical Cases. By C. C. F. Gay, M. D., .......... 301

### MISCELLANEOUS.

Against the Pendulum Movement, in Working the Midwifery Forceps, ..... 304
Effects Produced upon the Blood of Syphilitic Patients, by treatment with Mercury, .......... 308
Alteration of Sensibility in Articular Rheumatism, and the Electro-Therapeutical Treatment of the Disease, .......... 312

### EDITORIAL DEPARTMENT.

International Medical Congress, .......... 313
American Medical Association, .......... 315
Items, .......... 315
In Memoriam, .......... 317

BOOKS REVIEWED:

Medical Jurisprudence of Insanity. By J. H. Balfour Browne, Esq., . 317
Phthisis. By Austin Flint, M. D., .......... 318
A System of Midwifery. By William Lieshman, M. D., .......... 319

Books and Pamphlets Received, .......... 320

